# Therapeutic targeting of *BRCA1* and *TP53* mutant breast cancer through mutant p53 reactivation

**DOI:** 10.1038/s41523-019-0110-1

**Published:** 2019-04-15

**Authors:** Bing Na, Xin Yu, Tracy Withers, John Gilleran, Ming Yao, Tzeh Keong Foo, Chunxia Chen, Dirk Moore, Yong Lin, S. David Kimball, Bing Xia, Shridar Ganesan, Darren R. Carpizo

**Affiliations:** 1 Department of Surgery, Rutgers Robert Wood Johnson Medical School, New Brunswick, NJ USA; 2 Rutgers Cancer Institute of New Jersey, New Brunswick, NJ USA; 3 Rutgers University Biomedical Research Cores, Rutgers University, Piscataway, NJ USA; 4 Department of Medicinal Chemistry, Rutgers Ernest Mario School of Pharmacy, Rutgers University, Piscataway, NJ USA; 5 Department of Radiation Oncology, Rutgers Robert Wood Johnson Medical School, New Brunswick, NJ USA; 6 Department of Biostatistics, Rutgers School of Public Health, Rutgers University, New Brunswick, NJ USA; 7 Office of Innovation & Research Commercialization, Rutgers University, Piscataway, NJ USA; 8 Department of Pharmacology, Rutgers University, Piscataway, NJ USA; 9 Department of Medicine, Rutgers Robert Wood Johnson Medical School, New Brunswick, NJ USA; 10 Z53 Therapeutics, Inc., Holmdel, NJ USA

## Abstract

Triple negative breast cancer (TNBC) is an aggressive subset for which effective therapeutic approaches are needed. A significant proportion of TNBC patients harbor either germline or somatic mutations in *BRCA1*, or epigenetic silencing of *BRCA1*, which renders them deficient in DNA repair. Virtually all *BRCA1* deficient breast cancers harbor mutations in *TP53* suggesting that inactivation of p53 is a requirement for tumor progression in the setting of *BRCA1* deficiency. Due to this dependency, we hypothesized that restoring wild type p53 function in *BRCA1* deficient breast cancer would be therapeutic. The majority of *TP53* mutations are missense, which generate a defective protein that potentially can be targeted with small molecules. Zinc metallochaperones (ZMCs) are a new class of anti-cancer drugs that specifically reactivate zinc-deficient mutant p53 by restoring zinc binding. Using ZMC1 in human breast cancer cell lines expressing the zinc deficient p53^R175H^, we demonstrate that loss of BRCA1 sensitizes cells to mutant p53 reactivation. Using murine breast cancer models with *Brca1* deficiency, we demonstrate that ZMC1 significantly improves survival of mice bearing tumors harboring the zinc-deficient *Trp53*^*R172H*^ allele but not the *Trp53*^*−/−*^ allele. We synthesized a new formulation of ZMC1 (Zn-1), in which the drug is made in complex with zinc to improve zinc delivery, and demonstrate that Zn-1 has increased efficacy. Furthermore, we show that ZMC1 plus olaparib is a highly effective combination for p53^R172H^ tumor growth inhibition. In conclusion, we have validated preclinically a new therapeutic approach for *BRCA1* deficient breast cancer through reactivation of mutant p53.

## Introduction

Triple negative breast cancers (TNBC) are an aggressive subset of human breast cancer for which chemotherapy remains the standard treatment approach. *TP53* mutations are highly prevalent in TNBC, being present in almost all of these cancers.^1^ Approximately 10% of TNBC occur in patients harboring germline mutations in *BRCA1*, and additional subset of TNBC may harbor somatic mutations or epigenetic disruption of BRCA1 expression. Interestingly, almost all TNBCs that arise in the setting of germline *BRCA1* mutations harbor *TP53* mutations.^2–4^ These findings have suggested that inactivation of wild type p53 is a requirement for *BRCA1* mutant cancer development, and this notion is supported by mouse models where inactivation of p53 is required for efficient tumorigenesis in the setting of targeted *Brca1* loss.^5^ This is logical given that *BRCA1* or *BRCA2* deficiency increases genomic instability due to defects in double strand DNA break repair that require inactivation of cell cycle checkpoints like p53 for tumor initiation and progression.^6^ Although PARP inhibitors have been shown to be effective in the treatment of *BRCA1* mutant cancers, acquired resistance to these agents arises with treatment by several mechanisms including reversion, mutations and compensating mutations in other genes such as *TP53BP1*, limiting their efficacy.^7–10^ Thus, there is a pressing need to develop complementary, non-cross resistant therapeutic approaches for *BRCA1* mutant cancers.

The near universal dependence on p53 inactivation in *BRCA1* mutant tumor progression suggests that this relationship might be therapeutically exploited.^2,11^ Examples of approaches to target *TP53* mutations include inhibition of complementary cell cycle checkpoints mediated by WEE1 and Chk1, but these strategies only indirectly target p53 defect.^12,13^ A more attractive approach would be to directly restore wild type p53 function in a *BRCA1* deficient breast cancer. However, this approach remains untested due to the lack of clinically effective mutant p53 reactivators.

Zinc metallochaperones (ZMCs) are a new class of anti-cancer drugs that target a specific class of zinc-binding p53 mutations by restoring wild type p53 structure and function.^14,15^ Missense p53 mutants fall into three main categories: destabilizing, zinc-binding, and DNA contact.^16–18^ Zinc-binding mutants are classified by their proximity to the loops involved in coordinating the single zinc ion.^18^ This is best exemplified by the p53^R175H^, which is the most frequently found missense mutation in cancer.^19^ The substitution of a histidine for an arginine at codon 175 causes steric hindrance that weakens the affinity for zinc by approximately 100–1000 fold and thus at physiologic intracellular zinc concentrations the p53^R175H^ is in its apo-zinc free state and is misfolded.^20^ ZMCs raise intracellular zinc concentrations sufficiently high to allow zinc to ligate in the native binding site on p53^R175H^ and this permits proper folding.^21^

Here we explored the therapeutic relationship between *TP53* and *BRCA1* mutations in breast cancer using ZMCs as a tool to restore wild type p53 function. We hypothesized that BRCA1 loss renders cells highly sensitive to restoration of p53 function, and that restoring wild type p53 function in *BRCA1* mutant tumors could be a highly effective therapeutic strategy.

## Results

### Loss of BRCA1 sensitizes human tumor cells to mutant p53^R175H^ reactivation

**Fig. 1:**
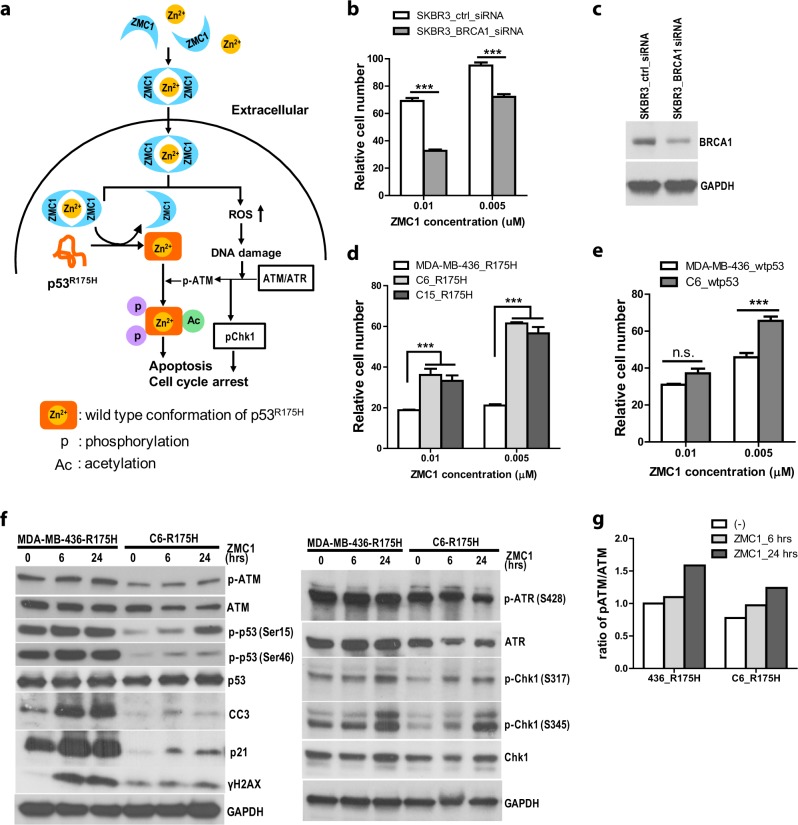
Loss of BRCA1 sensitizes cells to ZMC1. **a** Schematic representation of the mechanisms of ZMC1 reactivating mutant p53^R175H^. **b** The survival of breast cancer cell line SKBR3 (p53^R175H^) after knockdown with either control non-target siRNA or *BRCA1* siRNA. The cells were treated with 0.01 and 0.005 µM ZMC1. (*n* = 4) **c** Western blotting showed the BRCA1 knockdown by control siRNA (ctrl-siRNA) or BRCA1 siRNA (BRCA1-siRNA) in SKBR3 cells. **d** The survival of MDA-MB-436 and its two *BRCA1*-reconstituted cell lines (C6 and C15) after ZMC1 treatment. All the three cell lines were transiently transfected with plasmids expressing human mutant p53^R175H^ (R175H). (*n* = 4) **e** The survival of MDA-MB-436 and its *BRCA1*-reconstituted cell line (C6) after ZMC1 treatment. Both cell lines were transiently transfected with plasmids expressing human wild type p53 (wtp53). (*n* = 4) **f** Western blotting showed the proteins expression after 1 μM ZMC1 treatment for 6- and 24-hours in MDA-MB-436 and its *BRCA1*-reconstituted cell line (C6). Both cell lines were transiently transfected with plasmids expressing human p53^R175H^ (R175H). **g** Quantification of ATM and phospho-ATM (p-ATM) in (**f**). Student *t*-test; ***p < 0.001. Each survival experiment was repeated 2–3 times (Fig. 1b, d and e)

We have previously demonstrated that several structurally related members of the thiosemicarbazone family of metal ion chelators function to reactivate mutant p53 and as such we have classified them as ZMCs (ZMC1, 2, 3).^14,22^ We have also elucidated the mechanism of action of ZMC1 which is a two part mechanism. The first part involves the binding of zinc extracellularly in a 2:1 ratio of compound to zinc and freely diffusing across the plasma membrane as a zinc ionophore to deliver it to mutant p53 (Fig. 1a). The second part involves raising levels of cellular reactive oxygen species (ROS) through chelation of redox active metal ions like Fe^2+^, Cu^2+^. This leads to downstream signaling events in the DNA damage response (i.e., activation of ATM/ATR) which results in cell cycle arrest through phosphorylation of Chk1 as well as enhanced p53 signaling through the induction of post-translational modifications (PTMs) on mutant p53. These PTMs (phosphorylation on serines 15 and 46, acetylation on lysine-120) are normally associated with enhancing a p53 mediated apoptotic response (Fig. 1a).^20,23,24^ We hypothesized these downstream signaling events induced by ZMC1 would increase with the loss of BRCA1 which would render cells more sensitive to ZMC1 treatment.

SKBR3 is a human breast cancer cell line harboring p53^R175H^. We previously demonstrated that this cell line is highly sensitive to ZMC1^14^ and showed evidence of p53^R175H^ reactivation by ZMC1.^22^ To test the effect of BRCA1 loss in cells, we knocked down BRCA1 in SKBR3 cells using siRNA (Fig. 1c) and measured the cell survival upon ZMC1 treatment. We found that the cells transfected with the *BRCA1* siRNA had decreased survival upon ZMC1 treatment (Fig. 1b) and the decreased survival was not due to decreased growth of SKBR3 after BRCA1 knockdown (Supplementary Fig. S1A).

To confirm the effect of BRCA1 loss in ZMC1 sensitivity, we used a human TNBC cell line, MDA-MB-436, which is null for both BRCA1 and p53. This cell line has been previously reconstituted with BRCA1 by stable transfection and clones that express BRCA1 (Supplementary Fig. S1B) without an impact on cell growth have been characterized (C6 and C15) (unpublished data). We then transiently transfected plasmids expressing human p53^R175H^ into the parental and the complemented cell lines (Supplementary Fig. S1B) and compared their sensitivity to ZMC1. As predicted, the reconstituted cell lines, C6 and C15, had increased survival upon ZMC1 treatment (Fig. 1d), suggesting BRCA1 protected the cells from the ZMC1 effect.

We have previously shown that ZMC1 does not activate wild type p53 in tumor cells.^14^ We showed that tumor cells expressing endogenous levels of wild type p53 are relatively insensitive in addition to failing to induce p53 levels or upregulating p21. ZMC1 induces some degree of apoptosis (~25%) in tumor cells independent of p53 status, likely due to its ROS inducing effects. Nonetheless we sought to compare the contribution of wild type p53 versus mutant p53 in MDA-MB-436 and C6 with ZMC1 treatment by overexpressing both through transfection. As expected, cells expressing wild type p53 were less sensitive to ZMC1 than cells expressing the mutant (Fig. 1d and e). We also found that using 0.005 µM in both situations (expressing mutant or wild type p53) cells expressing reconstituted BRCA1 were less sensitive to ZMC1. These results indicate that ZMC1 is more effective in mutant p53 expressing cells versus wild type p53.

Due to the impact of the gain and loss of BRCA1 expression on the cell growth inhibition mediated by ZMC1, we sought to determine if this might be associated with differences in downstream signaling events in the MDA-MB-436 cell line series. Predictably, loss of BRCA1 also resulted in increased DNA double strand damage and decreased DNA repair as exemplified by the increased γH2AX in MDA-MB-436_p53^R175H^ cells compared to that of C6_p53^R175H^ after ZMC1 treatment (Fig. 1f left panel, g). In support of this, we evaluated these cells using the comet assay in response to the DNA damaging agent bleomycin as well as ZMC1. As predicted a significantly greater degree of DNA damage was observed in the MDA-MD-436_p53^R175H^ cells compared to that of C6_p53^R175H^ or the C15_p53^R175H^ cells (Supplementary Fig. 2b, d, e). Note that compared to bleomycin, ZMC1 induced DNA damage was significantly less (Supplementary Fig. S2A–C). In addition, baseline level of phosphorylated ATM was higher in the parental MDA-MB-436 cells than the BRCA1 complemented C6 clone, suggesting that there is increased baseline activation of DNA damage signaling in *BRCA1* mutant cells. Interestingly and consistent with this finding, we observed increased phosphorylation events on serines 15 and 46 on the p53^R175H^ in the parental MDA-MB-436 cells which was significantly reduced by re-introduction of BRCA1 (Fig. 1f left panel). Following p53 reactivation, markers of apoptosis (cleaved caspase-3 (CC3)) and cell cycle arrest (p21) increased (Fig. 1f left panel). On the other hand, DNA damage also triggers ATR activation, which in turn phosphorylates Chk1 (Fig. 1f right panel) contributing to cell cycle inhibition. Taken together, these data demonstrate loss of BRCA1 is associated with enhanced sensitivity to ZMC1. This is due to more robust activation of p53^R175H^ leading to greater apoptosis and enhanced signaling through the DNA damage response.

### ZMC1 reactivates mutant p53 in mouse models of *Brca1*-deficient breast cancer

**Fig. 2:**
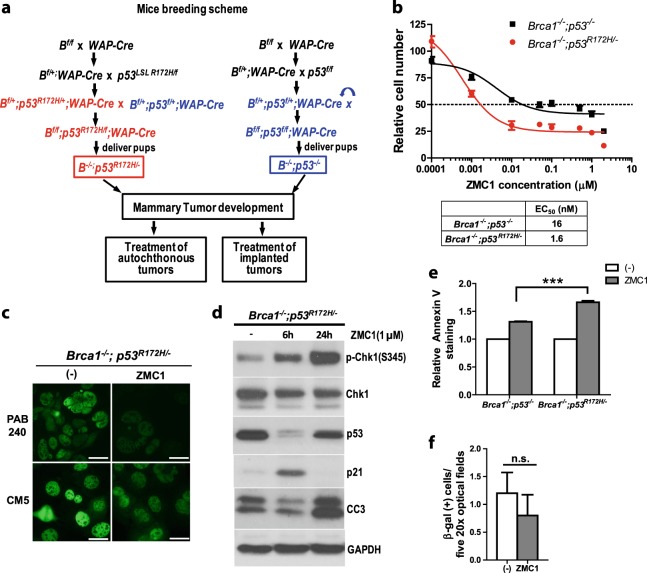
ZMC1 reactivates p53^R172H^ in *BRCA1-*deficient cell lines derived from genetically engineered mouse models. **a** Mice breeding scheme. **b** ZMC1 sensitivity of cell lines derived from *Brca1*^*−/−*^*;Trp53*^*−/−*^ and *Brca1*^*−/−*^ *Trp53*^*R172H/−*^ mouse breast tumors. (*n* = 4) **c** Immunofluorecence staining of the mutant p53^R172H^ protein in *Brca1*^*−/−*^ *Trp53*^*R172H/−*^ cells after 1 μM ZMC1 treatment for 6 h using PAB240 and CM5 antibodies. **d** Western blotting showed p-Chk1, p53, p21, and CC3 protein levels in *Brca1*^*−/−*^*;Trp53*^*R172H/−*^ cells after ZMC1 treatment. **e** Annexin V/PI staining showed the increase of apoptotic cells in *Brca1*^*−/−*^*;Trp53*^*−/−*^ and *Brca1*^*−/−*^*;Trp53*^*R172H/−*^ cells after 1 µM ZMC1 treatment for 24 h. Student *t*-test; ***p < 0.001. (*n* = 3) **f** β-galactosidase staining in *Brca1*^*−/−*^*;Trp53*^*R172H/−*^ cells after 0.5 μM ZMC1 treatment for 3 days. n.s.: not significant. (*n* = 5)

To evaluate the effects of p53 mutant reactivation in the setting of *Brca1* loss, we examined the effect of ZMC1 in mouse models of *Brca1*-deficient mammary cancer that harbor either the zinc-binding deficient p53^R172H^ (mouse equivalent of human p53^R175H^) or a p53-null mutation. We bred *WAP-Cre;Brca1*^*f/f*^*;Trp53*^*R172H/f*^ and *WAP-Cre;Brca1*^*f/f*^*;Trp53*^*f/f*^ mice by crossing *WAP-Cre* mice and *Brca1*^*f/f*^ mice with either *Trp53*^*LSL R172H/f*^ or *Trp53*^*f/f*^ mice. A diagram illustrating the breeding scheme and resultant genotypes is depicted (Fig. 2a). The *Brca1*^*f/f*^ allele corresponded to a deletion of exons 5–13, while the *Trp53*^*f/f*^ induced a deletion of exons 2–10, both confirmed by PCR (data not shown). These tumors exhibited similar histology with varied amount of stromas and there was no appreciable difference between the p53^R172H^ versus null tumors (Supplementary Fig. S3A). Both lines of mice displayed similar latency for mammary tumor development (Supplementary Fig. S3B).

Tumors from *WAP*-*Cre;Brca1*^*f/f*^*;Trp53*^*R172H/f*^ and *WAP-Cre;Brca1*^*f/f*^*;Trp53*^*f/f*^ mice were used to make primary tumor cell lines which were evaluated for cell survival in response to ZMC1. Presumably due to p53^R172H^ reactivation, we observed that the *Brca1*^*−/−*^; *Trp53*^*R172H*/−^ cells were significantly more sensitive to ZMC1 as compared to the *Brca1*^*−/−*^;*Trp53*^*−/−*^ (EC_50_ with 95% C.I. 1.6 nM (1.2, 2.1 nM) vs. 16 nM (8.7, 35.5 nM) (Fig. 2b). Using the mutant p53 specific PAB240 antibody, we found that treatment with ZMC1 induced a conformation change to wild type resulting in loss of PAB240 staining in the *Brca1*^*−/−*^;*Trp53*^*R172H*/−^ cells (Fig. 2c upper panels). We have previously shown that ZMC1 decreases mutant protein levels due to the restoration of MDM2 mediated negative autoregulation of p53.^14^ To validate the decreased PAB240 staining observed upon ZMC1 treatment was due to a conformation change and not merely due to MDM2 mediated degradation, we included CM5 antibody staining which recognizes both mutant and wild type conformations (Fig. 2c lower panels). Note that CM5 staining does decrease upon ZMC1 treatment in comparison to the untreated control (compare lower left panel to lower right panel) indicative of MDM2 mediated degradation. However, CM5 staining in the ZMC1 treated cells is greater than in the PAB240 ZMC1 treated cells indicating that a fraction of mutant p53 has undergone a WT conformation change (Fig. 2c compare right upper panel to right lower panel).

We recently determined that ZMC1’s mutant p53 reactivating activity (as shown by mutant p53 and p21 levels) in cells over 24 h is transient with the activity coming on by 6–8 h and is off by 24 h.^25^ Specifically mutant p53 levels decrease (and p21 levels increase) by 6–8 h and then return to baseline by 24 h. Furthermore, what turns off the function of ZMC1 is the induction of the cell’s zinc homeostatic mechanisms that normalize zinc levels. Once the levels of zinc normalized the mutant protein adopts its mutant conformation again and the drug is off.^25^ We then examined the *Brca1*^*−/−*^*;Trp53*^*R172H/−*^ cells for evidence of these transient pharmacodynamics and observed both a significant decrease in p53^R172H^ with a concomitant increase in p21 by 6 h that returned to baseline by 24 h (Fig. 2d).

It is not clear what cellular mechanism mutant p53 reactivation modulates in regards to cell death/survival in *Brca1*-deficient cells. To shed light on this, we examined these cells more carefully for evidence of apoptosis and senescence in response to ZMC1. For apoptosis, we found cleaved caspsase-3 levels increased continuously (Fig. 2d). We further looked for the percentage of apoptotic cells after ZMC1 treatment by Annexin V/PI staining and found that the apoptotic cells increased about 70% in *Brca1*^*−/−*^*;p53*^*R172H/−*^ cells upon ZMC1 treatment, in contrast to a 30% increase in *Brca1*^*−/−*^;*Trp53*^*−/−*^ cells (Fig. 2e and Supplementary Fig. S4C). Because activated p53 may induce cell senescence, we examined if ZMC1 treatment resulted in senescence of the *Brca1*^*−/−*^*;p53*^*R172H/−*^ cells. By β-galactosidase activity staining we confirmed that ZMC1 does not induce senescence (Fig. 2f). Taken together, these results substantiate the claim that the p53^R172H^ is being reactivated in the *WAP-Cre;Brca1*^*f/f*^ model and that ZMC1 has greater efficacy in *Brca1*^*−/−*^;*Trp53*^*R172H*/−^ cells as compared to *Brca1*^*−/−*^;*Trp53*^*−/−*^ cells.

### ZMC1 and a new formulation (ZMC1-zinc complex) improve survival in the *Brca1-*deficient mammary tumor GEMM in a *Trp53*^*R172H*^ allele specific fashion

**Fig. 3:**
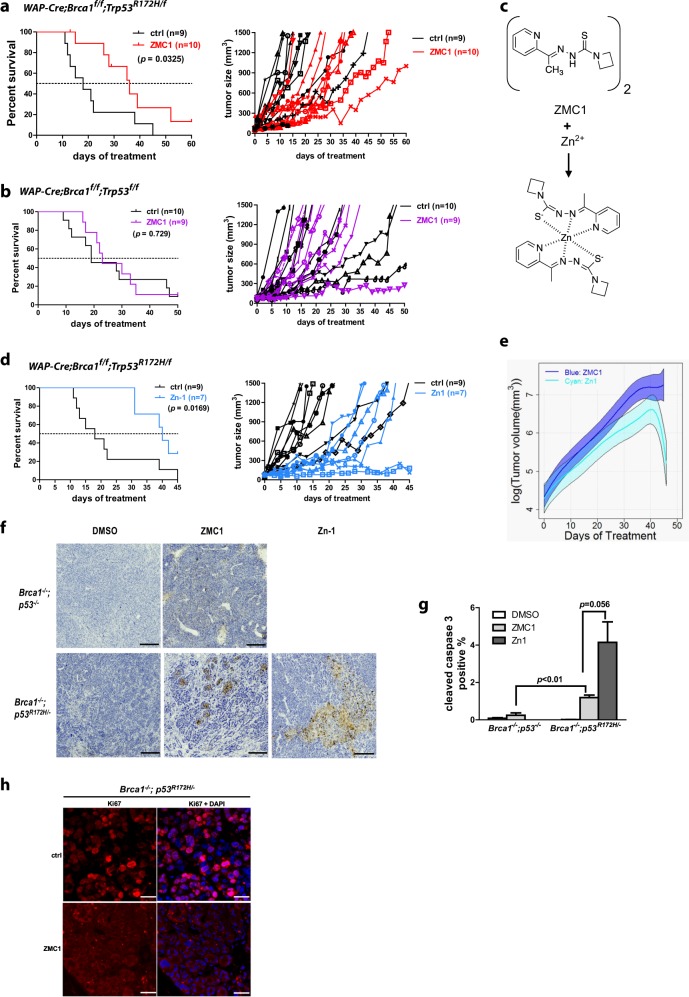
ZMC1 and its Zinc complex (Zn-1) improve the survival of *BRCA1*-deficeint mammary tumor bearing mice in a *Trp53*^*R175H*^ allele specific fashion. **a** Bearing *WAP-Cre;Brca1*^*f/f*^*;p53*^*R172H/f*^ mice were treated with either DMSO (ctrl, *n* = 9) or ZMC1 (2.5 mg/kg/d, *n* = 10). Kaplan-Meier survival curve (left panel) showed the overall survival of *WAP-Cre;Brca1*^*f/f*^*;p53*^*R172H/f*^ mice with DMSO or ZMC1 treatment. The individual tumor growth was shown in the right panel. **b** Tumor-bearing *WAP-Cre;Brca1*^*f/f*^*;Trp53*^*f/f*^ mice were treated with either DMSO (ctrl, *n* = 10) or ZMC1 (2.5 mg/kg/d, *n* = 9). Kaplan–Meier survival curve (left panel) showed the overall survival of *WAP-Cre;Brca1*^*f/f*^*;p53*^*f/f*^ mice with DMSO or ZMC1 treatment. The individual tumor growth was shown in the right panel. **c** The chemical structure of Zn-1. **d** Tumor-bearing *WAP-Cre;Brca1*^*f/f*^*;Trp53*^*R172H/f*^ mice were treated with DMSO (ctrl, *n* = 9) or Zn-1 (2.84 mg/kg/d, *n* = 7). Kaplan-Meier curve showed the overall survival of *WAP-Cre;Brca1*^*f/f*^;*Trp53*^*R172H/f*^ mice with DMSO or Zn-1 treatment (left panel). The individual tumor growth was shown in the right panel. **e** The average log tumor volume of ZMC1 (*n* = 10) or Zn-1 (*n* = 7) treated *WAP-Cre;Brca1*^*f/f*^*;Trp53*^*R172H/f*^ tumor-bearing mice versus post-injection time (in days). The fitted curves showed the log tumor volume of ZMC1 and Zn-1 treatment groups with the corresponding point-wise 84% confidence intervals (shaded bands). **f** Representative cleaved caspase-3 (CC3) staining in *Brca1*^*−/−*^*;Trp53*^*−/−*^ and *Brca1*^*−/−*^*;Trp53*^*R172H/−*^ tumors treated with either DMSO or ZMC1. **g** Quantification of CC3 in *Brca1*^*−/−*^*;Trp53*^*−/−*^ and *Brca1*^*−/−*^*;Trp53*^*R172H/−*^ tumors treated with either DMSO, ZMC1 or Zn-1 (three different tumors from three different tumor-bearing mice for each group). **h** Immunofluorescence staining of Ki67 in *Brca1*^*−/−*^*;Trp53*^*R172H/−*^ tumors treated with DMSO (ctrl) or ZMC1. Scale bar: 50 µm

To validate our in vitro results, we turned to study the efficacy of ZMC1 in vivo using the *WAP-Cre;Brca1*^*f/f*^*;Trp53*^*R172H/f*^ and *WAP-Cre;Brca1*^*f/f*^*;Trp53*^*f/f*^ mouse models. Starting from 4 months after the female *WAP-Cre;Brca1*^*f/f*^*;Trp53*^*R172H/f*^ and *WAP-Cre;Brca1*^*f/f*^*;Trp53*^*f/f*^ mice delivered their first litter, the mice were examined every 2–3 days for mammary tumor initiation. When tumors reached 50–100 mm^3^ in size, the mice were randomly assigned to either DMSO control group or ZMC1 treatment group (2.5 mg/kg/day by IP injection) and treated accordingly. We have conducted pre-clinical studies of ZMC1 to assess pharmacokinetics and acute toxicity and determined that ZMC1 exhibits a short half-life of about 30 min in mice and demonstrates a maximum tolerated dose of 5 mg/kg when administered IP in a pancreatic cancer GEMM.^25^ We found the MTD for this mammary tumor GEMM to be 2.5 mg/kg. Tumors were measured every 2–3 days, and as per institutional guidelines, mice were sacrificed if tumors reached 1500 mm^3^ and censored as a death. We found that ZMC1 treatment significantly delayed tumor development and improved survival in *WAP-Cre;Brca1*^*f/f*^*;Trp53*^*R172H/f*^ mice. The median survival time for the control group is 15 days, while the median survival time for the ZMC1-treated mice more than doubled at 35 days (*p* = 0.0325) (Fig. 3a left panel). ZMC1 had no effect on the survival of the *WAP-Cre;Brca1*^*f/f*^*;Trp53*^*f/f*^ mice (median survival control 19 days vs. ZMC1 23 days, *p* = 0.73) (Fig. 3b), indicating that the survival benefit of ZMC1 is *Trp53*^*R172H*^ allele specific.

We recently produced a new formulation of ZMC1, a drug:zinc complex, in which two molecules of ZMC1 are synthesized in complex with one molecule of zinc. This is based on several research findings we have made in the study of ZMC1: (1) adding supplemental zinc to the media containing ZMC1 improves its apoptotic function,^14^ and that X-ray crystal structure of ZMC1 in complex with zinc revealed a 2:1 stoichiometry.^23^ The chemistry to produce [Zn(ZMC1)_2_] (named Zn-1) is illustrated in Fig. 3c. We recently evaluated Zn-1 and demonstrated that it increased efficacy in vitro using a p53^R175H^ expressing ovarian cancer cell line in which Zn-1 treatment resulted in 100% cell kill by 3 days.^25^ In addition, Zn-1 demonstrated increased efficacy in vivo (over ZMC1 alone) in a pancreatic GEMM which expressed the p53^R172H^.^25^ We then sought to evaluate Zn-1 in the *WAP-Cre;Brca1*^*f/f*^*;Trp53*^*R172H/f*^ model. Similar to ZMC1 treatment, we treated the tumor-bearing *WAP-Cre;Brca1*^*f/f*^*;Trp53*^*R172H/f*^ mice with Zn-1 at 2.84 mg/kg/day and compared their growth with the vehicle treated control mice. The median survival time for the Zn-1-treatment group was 38 days, significantly longer than that of the control group (15 day) (*p* = 0.017) (Fig. 3d left panel).

To compare the response of both ZMC1 and Zn-1 in vivo, we measured tumor volumes of a single target lesion over time in each mouse where the target lesion was defined as the largest and most easily measurable tumor. A spider plot of the change in tumor volume over a 60 day time period demonstrates that the response to ZMC1 alone while effective in suppressing tumor growth in comparison to the vehicle control, was not able to induce stasis of tumor growth in any of the mice in the ZMC1 cohort (Fig. 3a right panel). Whereas with Zn-1, we observed a greater control over tumor growth in the entire Zn-1 cohort up to ~20 days in comparison to the vehicle control. In addition, we observed stasis of tumor growth out to 45 days in two mice (Fig. 3d right panel). To compare the response in tumor volume to both ZMC1 and Zn-1 in these mice, we performed an analysis using mixed effects models where the response was the log of tumor volume.^26^ The fitted tumor growth curves together with their point-wise 84% confidence intervals (CIs) were calculated so that if the two CIs did not overlap, then the difference in tumor volume between the treatment groups was statistically significant. This analysis revealed a significant reduction in tumor volume in the Zn-1 treated group as compared to the ZMC1 treated group by Day 42 (Fig. 3e). We have previously shown that CC3 immunostaining of treated tumor sections is a reliable biomarker of ZMC1 activity using a pharmacodynamics experiment in which mice were administered three doses of ZMC1 over 3 days and the tumors were harvested.^25^ CC3 staining was greater in ZMC1 treated *Brca1*^*−/−*^*;Trp53*^*R172H/−*^ tumors than *Brca1*^*−/−*^;*Trp53*^*−/−*^ tumors indicating an on target effect of ZMC1. Moreover, CC3 staining was greater in the Zn-1 treated *Brca1*^*−/−*^*;Trp53*^*R172H/−*^ tumors compared to ZMC1 treated tumors (Fig. 3f–g). In addition to increased apoptosis, we examined the cell proliferation using Ki-67 staining, and found that ZMC1 markedly decreased tumor cell proliferation (Fig. 3h).

### ZMC1 in combination with olaparib is highly effective for *Brca1*-deficient mouse mammary tumors

**Fig. 4:**
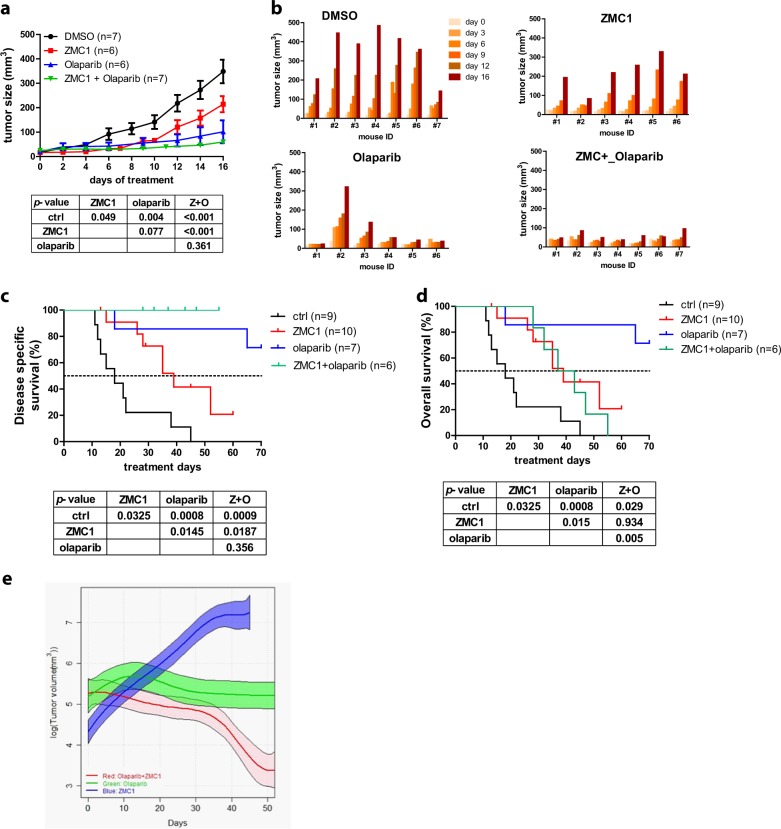
ZMC1 in combination with olaparib is a highly effective therapy for *Brca1*-deficient breast tumors. **a** Treatment efficacy of nude mice bearing implanted *Brca1*^*−/−*^*;p53*^*R172H/−*^ tumors with DMSO (*n* = 7), ZMC1 (*n* = 6), Olaparib (*n* = 6) or ZMC1 + Olaparib (*n* = 7). **b** Individual tumor growth with corresponding treatment as in (**a**). **c** Disease specific survival of mammary tumor-bearing *WAP-Cre*;*Brca1*^*f/f*^*;Trp53*^*R172H/f*^ mice treated with vehicle control (DMSO) (*n* = 9), ZMC1 (*n* = 10), olaparib (*n* = 7) or ZMC1 + olaparib (*n* = 6). **d** Overall survival of mammary tumor-bearing *WAP-Cre*;*Brca1*^*f/f*^*;Trp53*^*R172H/f*^ mice treated with DMSO (*n* = 9), ZMC1 (*n* = 10), olaparib (*n* = 7) or ZMC1 + olaparib (*n* = 6). **e** The average log tumor volume of *WAP-Cre;Brca1*^*f/f*^*;Trp53*^*R172H/f*^ tumor-bearing mice treated with ZMC1 (*n* = 10) olaparib (*n* = 7) or ZMC1 + olaparib (*n* = 6) versus post-injection time (in days). The fitted curves showed the log tumor volume of ZMC1, olaparib and ZMC1 + olaparib treatment groups and the corresponding point-wise 84% confidence intervals (shaded bands)

Olaparib, a poly ADP ribose polymerase (PARP) inhibitor, has recently been approved in the treatment of patients with BRCA1 and BRCA2 mutant breast cancer.^27^ Olaparib has been shown to be specifically toxic to BRCA1/2 mutant cells by generating DNA lesions, in part due to its PARP-trapping activity, that require homologous recombination for efficient repair. We hypothesized that induction of DNA lesions that are lethal to BRCA1 mutant cells by olaparib, coupled with the p53-reactivation activity of ZMC1, may be an effective combination in the *WAP-Cre;Brca1*^*f/f*^*;Trp53*^*R172H/f*^ model. Because the *Brca1*-deficient mammary tumor latency spans 4–12 months with mice developing multiple tumors with different growth kinetics, we initially sought to study this combination using a simpler tumor model in which the mice acquired tumors at relatively the same time, and the duration of therapy was relatively short. Therefore, we employed a model in which immunodeficient mice underwent orthotopic implantation (mammary fat pad) of fragments of tumor from each of the *WAP-Cre;Brca1*^*f/f*^*;Trp53*^*f/f*^ and *WAP-Cre;Brca1*^*f/f*^*;Trp53*^R172H/f^ tumor-bearing mice. When the implanted tumors reached 25–75 mm^3^ they were randomized to either vehicle control treatment, ZMC1 alone (5 mg/kg/day by IP injection), olaparib alone (50 mg/kg/day, by IP injection) or the combination of ZMC1 and olaparib. Upon treatment of the *Brca1*^*−/−*^;*Trp53*^*−/−*^ tumors with ZMC1, we observed a very mild and insignificant decrease in tumor growth (Supplementary Fig. S5A and B top panels) whereas in the mice bearing *Brca1*^*−/−*^;*Trp53*^*R172H*/−^ tumors, we observed a moderate and significant inhibition of tumor growth (Fig. 4a and b top panels, *p* = 0.049). This result was consistent with that obtained from the autochthonous *WAP-Cre;Brca1*-deficient model (Fig. 3), substantiating the conclusion that the anti-cancer efficacy of ZMC1 is specific to mutant p53^R172H^. Olaparib was very effective as a single agent in mice bearing *Brca1*^*−/−*^;*Trp53*^*R172H*/−^ tumors (Fig. 4a, *p* = 0.004). The combination of olaparib and ZMC1 showed a slight improvement in survival that was not statistically significant (Fig. 4a, *p* = 0.361). Moreover, tumor growth kinetics displayed in each individual mouse demonstrated stasis of tumor growth in 5/7 mice (Fig. 4b lower panels). Upon treatment of mice bearing *Brca1*^*−/−*^;*Trp53*^*−/−*^ tumors with olaparib, we also observed a potent inhibition of tumor growth. The addition of ZMC1 in this cohort also showed no improved efficacy (Supplementary Fig. S5). Interestingly this potent tumor growth inhibition was difficult to demonstrate in vitro as when we treated *Brca1*^*−/−*^;*Trp53*^*R172H*/−^ and *Brca1*^*−/−*^;*Trp53*^*−/−*^ cells with up to 50 μM, we found the cell lines were relative resistant and showed only mild increases in pChk1, p21 and CC3 after 6 h that decreased by 24 h (Supplemental Fig. S4A and B).

Because tumor growth in this model was fairly rapid and the duration of treatment was relatively short (16 days), we suspected that the combination of olaparib and ZMC1 may have a greater effect in the autochthonous model where tumor growth is slower and duration of treatment is longer (50–70 days). Treatment of *WAP-Cre;Brca1*^*f/f*^*;Trp53*^*R172H/f*^ mice with olaparib alone was well tolerated with no toxicity and led to a significant improvement in disease-specific survival (DSS) whereby 70 days of treatment 75% of the mice were alive (Fig. 4c). When we added ZMC1 to olaparib, we found that the combination exhibited toxicity in mice that manifested clinically as generalized weight loss. This toxicity was most evident after a period of 20 days of treatment and required holding treatment for 1–5 days in four of six mice. Despite this, none of the mice in this cohort died or were sacrificed because of tumor progression and as such they exhibited 100% disease specific survival with 5/10 mice surviving beyond the median survival of ZMC1 alone before being sacrificed or dying of toxicity (Fig. 4c). When we examined the Kaplan–Meier survival curve using overall survival as the endpoint, we observed that the combination was slightly better than ZMC1 and significantly worse than olaparib alone, due to toxicity of the combined regimen (Fig. 4d). We next compared the tumor growth (Supplementary Fig. S6) in log tumor volume using the fitted tumor growth curves (Fig. 4e) as in Fig. 3e. We found that, with a significance level of 5%, the difference in tumor volumes between ZMC1 and olaparib + ZMC1 was statistically significant after around Day 15, the difference between ZMC1 and olaparib was statistically significant after around Day 22, and the difference between olaparib and olaparib + ZMC1 was significant after around Day 36. Thus, by this measure, the combination of olaparib and ZMC1 produced a significantly greater response compared to olaparib alone.

## Discussion

Here for the first time we have demonstrated pre-clinically that reactivating mutant p53 in the setting of *Brca1* deficient breast cancer is highly therapeutic. This provides evidence supporting the concept that the dependency on loss of *TP53* in *BRCA1* mutant human breast cancers can be exploited therapeutically. What currently limits the ability to translate this clinically is the availability of mutant p53 reactivating drugs which have only recently been introduced into the clinic; however, none are approved therapies. APR-246, a small molecule mutant p53 reactivator is currently leading the way and is being studied in five clinical trials (phase 1–2) in various solid organ cancers (ovarian, esophagus, melanoma) but none in breast cancer. ZMCs are still in pre-clinical development, but other approaches to target this dependency indirectly are being investigated. One such approach is using cell cycle checkpoint inhibitors such as WEE1 or Chk1 inhibitors in combination with DNA damaging agents (i.e., platinum) or PARP inhibitors in *BRCA1* deficient breast cancer. This is an attractive approach as it theoretically would be useful for tumors with both missense and null *TP53* mutations.

ZMCs are distinguished from other mutant p53 reactivators in that their activity is limited to a specific subset of p53 missense mutations that impair the protein’s ability to bind zinc. This pertains to mutants involving the four amino acids that directly coordinate the zinc ion (C176, H179, C238, and C242) and also pertains to mutants at codons that are in close proximity to the zinc binding site (R175, for instance); however, the full spectrum of these mutations is currently unknown. We now have evidence of a larger range of mutations that are amenable to reactivation with ZMCs that are not only zinc binding mutants but also destabilizing hot spot mutants like p53^G245S^.^20^ Thus the potential pool of patients amenable to this new class of drugs is growing. This is an attractive aspect to the development of ZMCs because the patient population in whom to enroll in future clinical trials is known (those with zinc deficient p53 mutant tumors). This study would go further to identify a subgroup of patients that would likely respond to ZMCs, which is breast cancer patients that harbor *BRCA1* mutations. We speculate that this would also apply to other tumor types in which *BRCA1* mutations are found such as ovarian and pancreas.

PARP inhibitors represent an important advancement in the treatment of *BRCA1* deficient breast cancers; however, resistance is now a well-recognized problem. Resistance can occur both by reversion mutations, and by compensating mutations in TP53BP1 and related proteins, such as REF7 and RIF1, that can partly restore DNA repair capacity. Acquired resistance to PARP inhibitors. This highlights the need to identify new combinations with PARP inhibitors that can prevent or minimize acquired resistance. We found that the combination of olaparib and ZMC1 appears to be quite effective, although toxic with chronic therapy at tested doses in the *WAP-Cre;Brca1*^*f/f*^*;Trp53*^*R172H/f*^ model. This model is notoriously difficult to use in pre-clinical therapeutic experiments due to its long latency and multiplicity of tumors with different tumor kinetics in the same animal. Thus optimizing the dose/schedule of each agent to achieve a well-tolerated combination would take significant periods of time and this was not the main purpose of this study. Nonetheless, the 100% DSS survival benefit produced by the combination certainly is intriguing and warrants further investigation to identify a dose/schedule that is safe. We speculate that this combination may provide an approach to prevent or impair the acquired resistance to PARP inhibitors by allowing treatment breaks in which each agent may be alternately administered.

## Methods

### Chemicals, cell lines, and culture conditions

Olaparib was purchased from Selleckchem (Houston, TX). SKBR3 cell line was purchased from American type cell culture (ATCC) and cultured in McCoy’s 5A medium. MDA-MB-436 (purchased from ATCC), C6, C15, and mouse tumor derived cell lines were cultured in DMEM/F12 medium. All the culture media were supplemented with 10% FBS and 100 µg/ml penicillin/streptomycin.

ZMC1 and Zn-1 were synthesized by the Rutgers Translational Sciences group. Zn-1 was synthesized as follows: ZnCl_2_ (55.5 mg, 0.407 mmol, 0.5 equiv.) was added to a suspension of ZMC1 (190.8 mg, 0.814 mmol, 1 equiv.) in EtOH (20 ml). After 5 minutes, TEA (0.80 ml, excess) was added and the mixture was heated for 2 h at reflux under nitrogen. Upon cooling to ambient temperature, a solid precipitated that was collected by filtration and washed with EtOH followed by Et_2_O. The solids were dried under high vacuum to give Zn-1 (215 mg, 0.404 mmol, 99%) as a bright yellow solid. 1H-NMR (400 MHz, DMSO-d6) δ 2.26 (overlapping tt, J = 7.48 Hz, 7.40 Hz, 4 H), 2.58 (s, 6 H), 4.05 (m, 8 H), 7.29 (dd, J = 7.28 Hz, 5.60 Hz, 2 H), 7.75 (m, 4 H), 7.88 (dt, J = 8.04 Hz, 1.52 Hz, 2 H). Slow evaporation of Zn-1 from a 1:1 mixture of DCM/MeOH afforded yellow crystals that were suitable for X-ray crystallography.

### Cell survival assay

Cell survival was measured using MTS assay following manufacturer’s instruction. Five thousand cells were seeded in 96-well plates and treated with ZMC1 of various concentration for 3 days. MTS reagent was added to the cell culture and the cells continued to incubated for 1 h at 37 °C. The absorbance of the culture was measured using TECAN Infinite 200 PRO plate reader at 490 nm.

### Transfection of siRNA and plasmids

Human *BRCA1* siRNA was custom synthesized by Dharmacon. Two custom *BRCA1* siRNA sequences were employed: GGAACCUGUCUCCACAAAGdTdT and GCAGAUAGUUCUACCAGUAdTdT. Twenty-five pmol of siRNA was transfected into 8 × 10^5^ SKBR3 cells using 7.5 µl of Lipofectamine RNAiMAX reagent (Invitrogen) according to the manufacturer’s protocol. Control cells were transfected with Dharmacon’s ON-TARGETplus non-targeting siRNA. Cells treated with ZMC1 or vehicle were collected two days after transfection. Plasmids expressing human mutant p53^R175H^ or wild type p53 were transfected into MDA-MB-436 or its derived cells using Lipofectamine 2000 (Invitrogen).

### Comet assay

Comet assay was performed using CometAssay kit (Trevigen) following the manufacturer’s protocol. Briefly, trypsinized cells were suspended in PBS (Ca^2+^ and Mg^2+^ free) at 5 × 10^5^ cells/ml. Ten microliter of the cells were mixed with 100 µl of 37 °C LMAgarose and 50 µl of the mixture were transferred onto a prewarmed (37 °C) CometSlide. The slides were immersed in Lysis Solution at 4 °C overnight and then in Alkaline Unwinding Solution (200 mM NaOH, 1 mM EDTA) for 20 min at room temperature. The slides were subjected to electrophoresis in Alkaline Electrophoresis Solution (200 mM NaOH, 1 mM EDTA) for 30 min at 21 volts in a CometAssay ES unit. The slides were washed in dH_2_O for 5 min twice and once in 70% ethanol and then dried at 37 °C for 10–15 min. Place 100 µl of diluted SYBR green onto each slide and stain for 30 min at room temperature in dark. Allow slides to dry completely at 37 °C. The fluorescent images were analyzed using KEYENCE BZ-X710 Fluorescence Microscope.

### Western blotting

Cells were lysed in RIPA buffer (Thermo Fisher Scientific) supplemented with 1x protease inhibitor cocktail (Sigma) and 1x Halt Phosphatase Inhibitor Cocktail (ThermoFisher Scientific). Protein concentration was determined using Coomassie Plus (Bradford) assay kit (Thermo Fisher Scientific). Twenty microgram of protein samples were subjected to SDS-PAGE and probed by appropriate antibodies. The following antibodies were purchased from Cell Signaling Technology for WB: human p21 (#2947, 1:1000), ATM (#2873, 1:1000), phospho-ATM (#5883, 1:1000), ATR (#2790, 1:1000), phospho-ATR (#2853, 1:1000), phospho-Chk1 (Ser345) (#2348, 1:1000), phospho-Chk1(Ser317) (#13202, 1:1000), human phospho-p53 (Ser15) (#9284,1:1000), human phospho-p53 (Ser46, #2521) (#9284,1:1000), CC3 (#9664, 1:1000), anti-BRCA1 (#9010, 1:1000), Phospho-Histone H2A.X (Ser139) (#9718, 1:1000). Other antibodies: mouse p53 (Leica Biosyst, NCL-L-p53-CM5p, 1:4000), human p53 (Santa Cruz, sc-126, 1:3000), anti-mouse p21 (Santa Cruz, sc-6246, 1:500), Chk (Bethyl Laboratory, A300-161A, 1:3000), anti-GAPDH (Santa Cruz, sc-25778, 1:4000).

All the samples for the same experiment were prepared at the same time. Although different blots may be processed at different time, samples on the same blot (they are compared to each other) were processed at the same time.

### Establishment of mouse mammary tumor cell lines

Mouse mammary tumors were isolated and cut into small pieces. A tiny piece of the tumor was minced and passed through a 70 μm sterile cell strainer and seeded in a 10-cm dish with DMEM/F12 medium supplemented with 10% FBS and 100 µg/ml penicillin/streptomycin. The medium was changed the next day and every 3–4 days thereafter till single cells formed isolated colonies. The single colonies were digested with 0.05% Trypsin and transferred to 96-well plates. The individual cell clones were expanded to 10-cm petri dishes and the stocks were made for future use.

### Annexin V staining

Twenty thousand cells were seeded in 12-well plates and treated with 1 µM ZMC1 for 24 h. Cells were then trypsinized and resuspended in the original medium. Mix 100 µl of the resuspended cells with 100 µl of Guava Nexin Reagent (Millipore) in a 96-well plate and subject to Guava EasyCyte Flow Cytometer.

### β-galactosidase staining

Cells were seeded in 6-well plates at 2 × 10^5^/well and treated with 0.1 µM ZMC1 for 3 days. Their β-galactosidase activity was stained using Senescence β-Galactosidase Staining Kit (Cell Signaling Technology, #9860) following manufacturer’s protocol. Positive cells were counted in five randomly chosen fields under 20× light microscope. The numbers were averaged and plotted.

### Animal experimentation

All animal experiments were conducted in accordance with the protocols approved by the Institutional Animal Care and Use Committee (IACUC) of Rutgers Robert Wood Johnson Medical School. Female *WAP-Cre;Brca1*^*f/f*^*;Trp53*^*f/f*^ and *WAP-Cre;Brca1*^*f/f*^*;Trp53*^*R172H/f*^ were obtained by crossing *WAP-Cre* mice ((O1XA8, B6.Cg-Tg(Wap-Cre)11738Mam/Nci and^28^) and Brca1^*f/f*5^ mice with either *Trp53* LSL 172H (O1XM2, 129S4-*Trp53*^tm2Tyj^/Nci) or Trp53^f/f^.^29^ The floxed *Brca1*, floxed *Trp53*, *Trp53 LSL* and *WAP-Cre* were determined by PCR. Female mice with correct genotypes underwent pregnancy and lactating before they were observed for mammary tumor development. The female mice were examined for tumor occurrence every 2–3 days 4 months after they delivered the pups. Tumor sizes were determined by measuring the tumor lengths and widths using a caliper and calculated following the formula: (length (mm) × width (mm) × width (mm) × 3.14)/6. ZMC1 and Zn-1 were dissolved in DMSO for intraperitoneal (IP) administration to the transgenic mice at 2.5 mg/kg/day and 2.84 mg/kg/day respectively. For the xenograft mouse model ZMC1 was applied at 5 mg/kg/day by IP injection. Olaparib was dissolved in DMSO and administered to the mice at 50 mg/kg/day by IP injection.

### Quantification of cleaved caspase-3

Specimens were digitized at 20x with Olympus VS120 whole slide scanner (Olympus Corporation of the Americas). Image analysis protocol was custom developed on Visiopharm image analysis platform (Visiopharm A/S) to identify specifically stained cells/particles and compute its area burden on affected tissue.

### Statistics

Tumor volume analyses were performed based on the log of tumor volume in order to satisfy the model assumptions. Mixed effect models^26^ were used in the analysis, where the response was the log of tumor volume, the cubic spline (with three knots evenly distributed between 0 and 50 days)^30^ was used to estimate tumor growth trends, and the random intercept was included to account for the within-mouse correlation. The model was fitted separately for each treatment. The fitted tumor growth curves together with their point-wise 84% confidence intervals (CIs) were calculated so that if the two CIs were not overlapping, the difference in tumor volume between the two corresponding treatment groups at the time point is statistical significant at significance level of ~5%.^31,32^ All the analyses were performed using R statistical system version 3.2.5 (R Foundation for Statistical Computing, Vienna, Austria) with packages nlme version 3.1–125 and spline version 3.2.5.

### Reporting summary

Further information on experimental design is available in the Nature Research Reporting Summary linked to this article.

## Supplementary information

Supplementary figures

Reporting Summary

## Data Availability

The statistical data generated and analyzed during this study are described in the following data record^33^: 10.6084/m9.figshare.7688537. As described in the data record, data are available from the authors on request. The western blot results have associated raw data that are available in Supplementary Information.

## Supplementary information

**Supplementary information** accompanies the paper on the *npj Breast Cancer* website (10.1038/s41523-019-0110-1).

## References

[1] Cancer Genome Atlas, N. (2012). Comprehensive molecular portraits of human breast tumours. Nature.

[2] Holstege H (2009). High incidence of protein-truncating TP53 mutations in BRCA1-related breast cancer. Cancer Res.

[3] Greenblatt MS, Chappuis PO, Bond JP, Hamel N, Foulkes WD (2001). TP53 mutations in breast cancer associated with BRCA1 or BRCA2 germ-line mutations: distinctive spectrum and structural distribution. Cancer Res.

[4] Gasco M, Yulug IG, Crook T (2003). TP53 mutations in familial breast cancer: functional aspects. Hum. Mutat..

[5] Liu X (2007). Somatic loss of BRCA1 and p53 in mice induces mammary tumors with features of human BRCA1-mutated basal-like breast cancer. Proc. Natl Acad. Sci. USA.

[6] Narod SA, Foulkes WD (2004). BRCA1 and BRCA2: 1994 and beyond. Nat. Rev. Cancer.

[7] Henneman L (2015). Selective resistance to the PARP inhibitor olaparib in a mouse model for BRCA1-deficient metaplastic breast cancer. Proc. Natl Acad. Sci. USA.

[8] Kim Y (2017). Reverse the Resistance to PARP Inhibitors. Int. J. Biol. Sci.

[9] Jaspers JE (2013). Loss of 53BP1 causes PARP inhibitor resistance in Brca1-mutated mouse mammary tumors. Cancer Discov..

[10] Ganesan, S. Tumor suppressor tolerance: reversion mutations in BRCA1 and BRCA2 and resistance to PARP inhibitors and platinum. *JCO-Precis. Oncol.* **2** (2018). 10.1200/PO.18.00001 35135103

[11] Schuyer M, Berns EM (1999). Is TP53 dysfunction required for BRCA1-associated carcinogenesis. Mol. Cell. Endocrinol.

[12] Chen Z (2006). Selective Chk1 inhibitors differentially sensitize p53-deficient cancer cells to cancer therapeutics. Int. J. Cancer.

[13] Hirai H (2009). Small-molecule inhibition of Wee1 kinase by MK-1775 selectively sensitizes p53-deficient tumor cells to DNA-damaging agents. Mol. Cancer Ther..

[14] Yu X, Vazquez A, Levine AJ, Carpizo DR (2012). Allele-specific p53 mutant reactivation. Cancer Cell.

[15] Blanden AR, Yu X, Loh SN, Levine AJ, Carpizo DR (2015). Reactivating mutant p53 using small molecules as zinc metallochaperones: awakening a sleeping giant in cancer. Drug Discov. Today.

[16] Bullock AN, Fersht AR (2001). Rescuing the function of mutant p53. Nat. Rev. Cancer.

[17] Joerger AC, Fersht AR (2007). Structure-function-rescue: the diverse nature of common p53 cancer mutants. Oncogene.

[18] Joerger AC, Ang HC, Fersht AR (2006). Structural basis for understanding oncogenic p53 mutations and designing rescue drugs. Proc. Natl Acad. Sci. USA.

[19] Freed-Pastor WA, Prives C (2012). Mutant p53: one name, many proteins. Genes Dev..

[20] Yu X (2014). Small molecule restoration of wildtype structure and function of mutant p53 using a novel zinc-metallochaperone based mechanism. Oncotarget.

[21] Butler JS, Loh SN (2003). Structure, function, and aggregation of the zinc-free form of the p53 DNA binding domain. Biochemistry.

[22] Yu X (2017). Thiosemicarbazones functioning as zinc metallochaperones to reactivate mutant p53. Mol Pharmacol.

[23] Blanden AR (2015). Synthetic metallochaperone ZMC1 rescues mutant p53 conformation by transporting zinc into cells as an ionophore. Mol. Pharmacol.

[24] Smith J, Tho LM, Xu N, Gillespie DA (2010). The ATM-Chk2 and ATR-Chk1 pathways in DNA damage signaling and cancer. Adv. Cancer Res..

[25] Yu X (2018). Zinc metallochaperones reactivate mutant p53 using an ON/OFF switch mechanism: a new paradigm in cancer therapeutics. Clin. Cancer Res..

[26] Diggle, P. J, Heagerty, P., Liang, K. Y. & Zeger, S. L. *Analysis of Longitudinal Data*, 2nd edn. (Oxford University Press, Oxford, 2005).

[27] Robson M (2017). Olaparib for metastatic breast cancer in patients with a germline BRCA mutation. N. Engl. J. Med..

[28] Wagner KU (1997). Cre-mediated gene deletion in the mammary gland. Nucleic Acids Res..

[29] Jonkers J (2001). Synergistic tumor suppressor activity of BRCA2 and p53 in a conditional mouse model for breast cancer. Nat. Genet..

[30] Harrel, F. E. *Regression Modeling Strategies: With Application to Linear Models, Logistic and Ordinal Regression, and Survival Analysis*, 2nd edn. (Springer Series in Statistics, Springer, Berlin, Germany, 2015).

[31] Julious SA (2004). Using confidence intervals around individual means to assess statistical significance between two means. Pharmaceutical Statistics.

[32] Cumming G (2009). Inference by eye: Reading the overlap of independent confidence intervals. Statistics in Medicine.

[33] Na B (2019). Supporting metadata for Therapeutic targeting of BRCA1 and TP53 Mutant Breast Cancer Through Mutant p53 reactivation. Figshare.

