# Supplementary material for: Therapeutic targeting of BRCA1 and TP53 mutant breast cancer through mutant p53 reactivation
Source: NPJ Breast Cancer. 2019 Apr 15;5:14. doi: 10.1038/s41523-019-0110-1 (PMC6465291; doi:10.1038/s41523-019-0110-1)
Supplement: Supplementary file 1 — Supplementary figures [file 41523_2019_110_MOESM1_ESM.pdf]

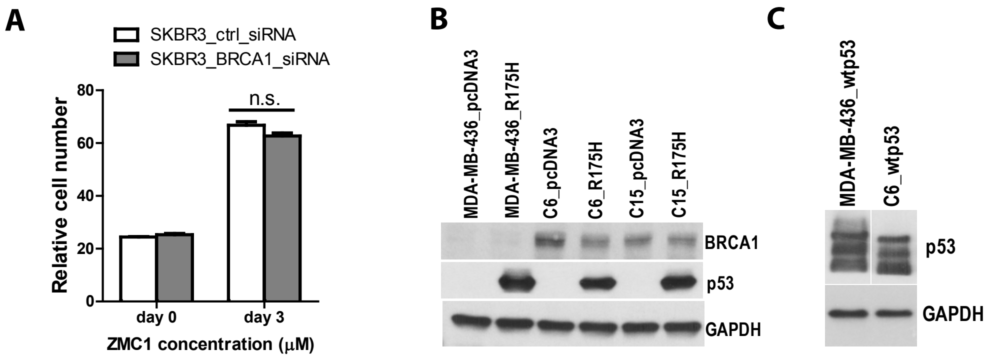

Supplementary Figure S1 (A) The growth inhibition effect of ZMC1 on SKBR3 transfected with control non-target or *BRCA1* siRNA knock down. (B) Western blot showed BRCA1 and $p53^{R175H}$ (R175H) expression in MDA-MB-436 and its BRCA1 reconstituted cell lines (C6 and C15). (C) Western blotting confirmed the wild type p53 expression in MDA-MB-436 and C6.

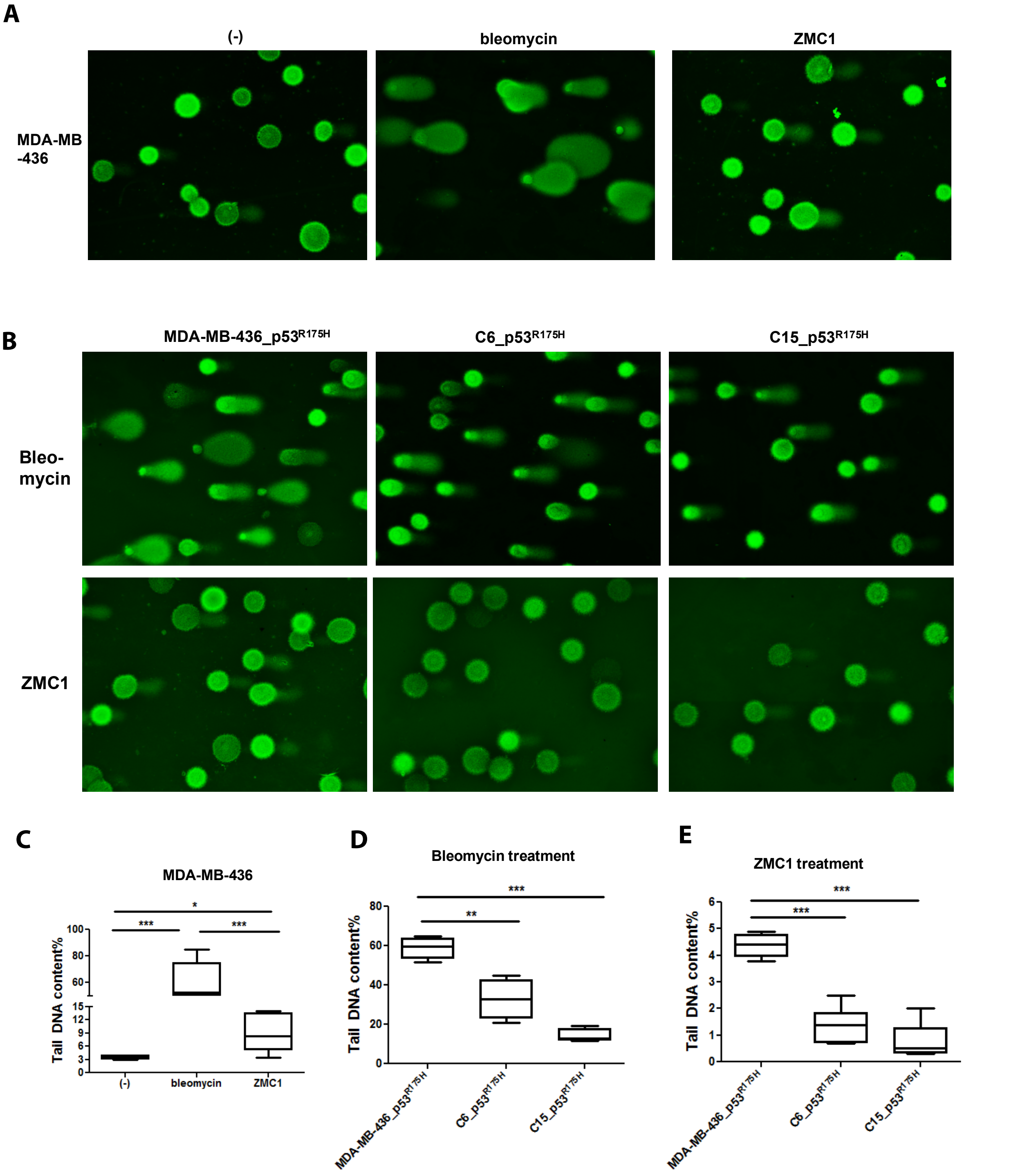

Supplementary Figure S2 Comet assay shows the DNA damage in MDA-MB-436 and its derived cells. (A) DNA damage of MDA-MB-436 treated with 30 μM bleomycin for 2 hours and 1 μM ZMC1 for 6 hours. (B) The DNA damage of MDA-MB-436_p53^{R175H}, C6_p53^{R175H} and C15_p53^{R175H} treated with 30 μM bleomycin for 2 hours (upper panels) or 1 μM ZMC1 for 6 hours (lower panels). (C) Quantification of DNA damage in (A). (D) Quantification of DNA damage by 30 μM bleomycin for 2 hours in (B) upper panels. (E) Quantification of DNA damage by 1 μM ZMC1 for 6 hours in (B) lower panels. Student *t*-test; *: $p<0.05$; **: $p<0.01$; ***:$p<0.001$.

A

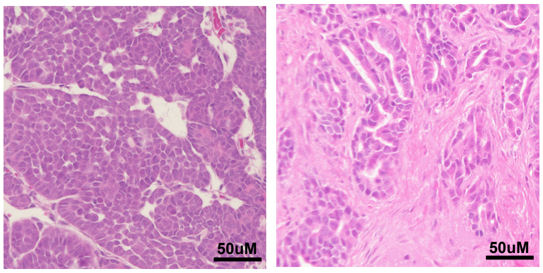

B

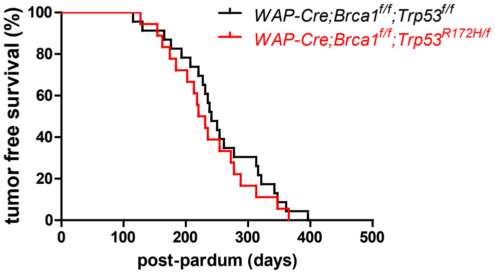

Supplementary Figure S3 *WAP-Cre;Brca1*$^{f/f}$*;p53*$^{R172H/f}$ and *WAP-Cre;Brca1*$^{f/f}$*;p53*$^{f/f}$ mice developed mammary tumors. (A) Representative histopathology of *WAP-Cre;Brca1*$^{f/f}$*;p53*$^{R172H/f}$ and *WAP-Cre;Brca1*$^{f/f}$*;p53*$^{f/f}$ mammary tumors by H&E staining. (B) Kaplan-Meier plot shows tumor free survival of *WAP-Cre;Brca1*$^{f/f}$*;p53*$^{R172H/f}$ and *WAP-Cre;Brca1*$^{f/f}$*;p53*$^{f/f}$ mice.

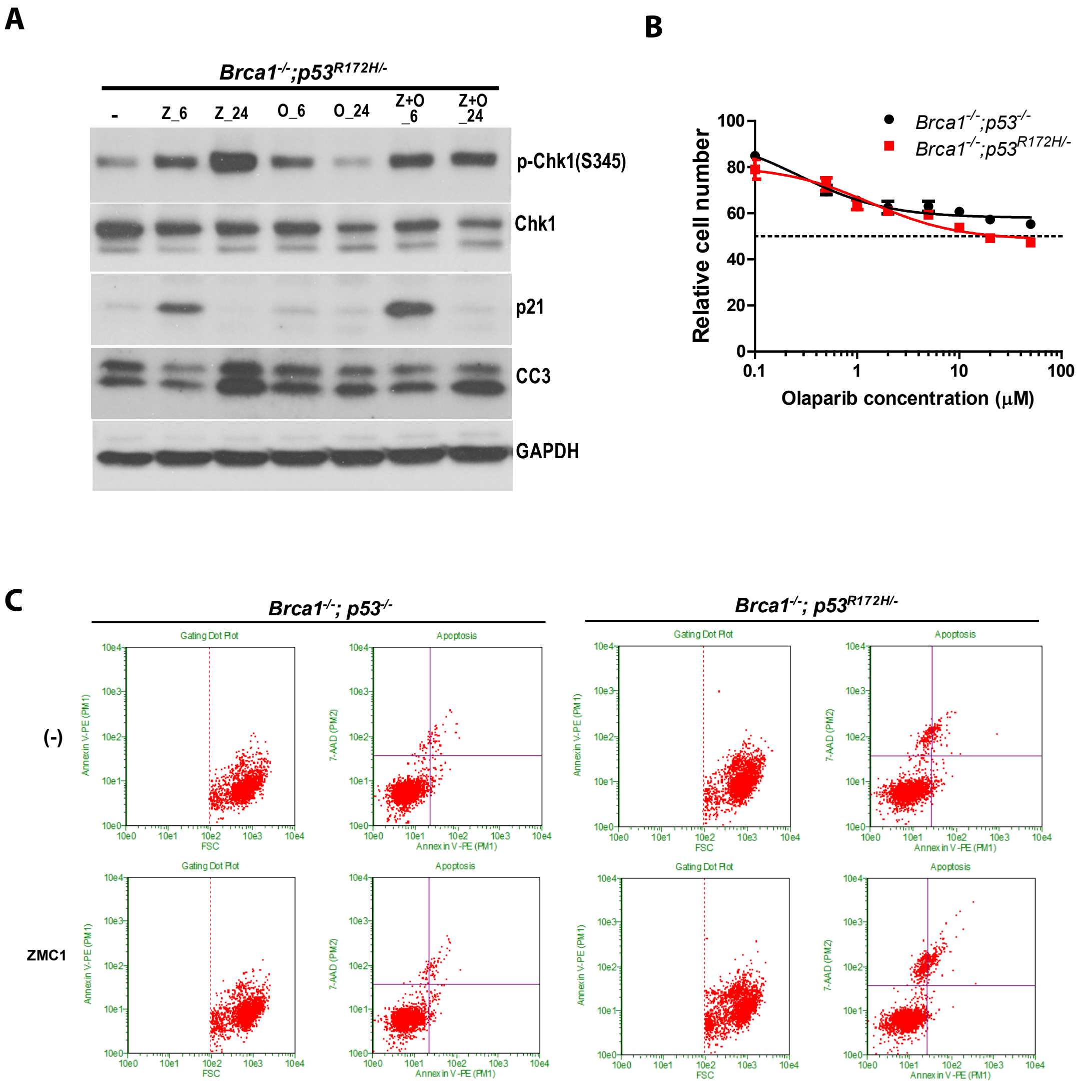

Annexin V (+)%	(-)	ZMC1
B$^{-/-}$ *;p53*$^{-/-}$	4.0	5.2
B$^{-/-}$ *;p53*$^{R172H/-}$	7.9	12.7

Supplentary Figure S4 (A) Western blotting showed the induction of p-Chk1, p21 and CC3 afte6- and 24-hour treatment of ZMC1(Z, 1 μM), orlaparib (O, 50 μM) or ZMC1+olaparib (Z+O) in the *Brca1*$^{-/-}$*;Trp53*$^{-/-}$ and *Brca1*$^{-/-}$*;Trp53*$^{R172H/-}$ cells. 6: 6-hr treatment; 24: 24-hr treatment. (B) Survival of *Brca1*$^{-/-}$*;Trp53*$^{-/-}$ and Brca1$^{-/-}$;Trp53$^{R172H/-}$ cells with olaparib treatment. (C) Flow cytometry result of Annexin V/PI staining of *Brca1*$^{-/-}$*;p53*$^{-/-}$ and *Brca1*$^{-/-}$*;p53*$^{R172H/-}$ cells treated with ZMC1.

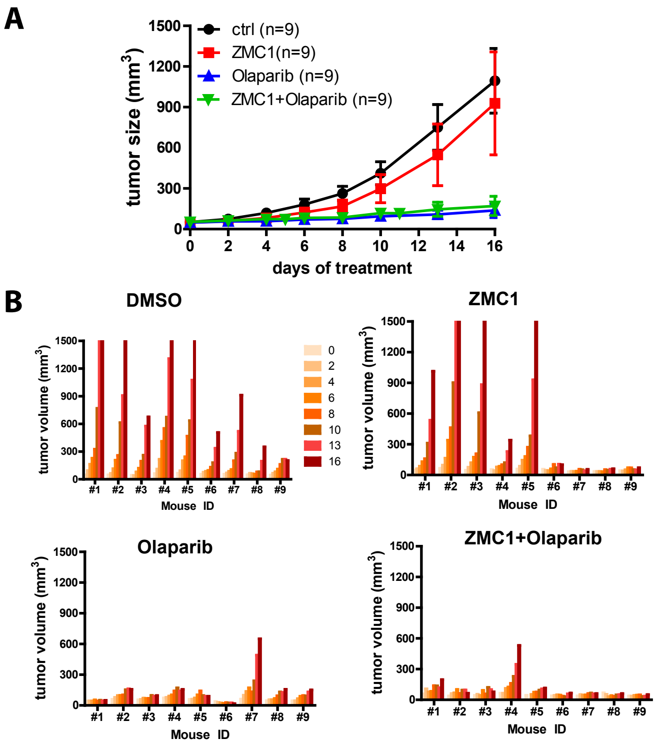

Supplementary Figure S5 Treatment of nude mice bearing implanted *Brca1*$^{-/-}$;*p53*$^{-/-}$ breast tumor fragments with either DMSO, ZMC1, olaparib or ZMC1+olaparib. (A) Nude mice carrying implanted *Brca1*$^{-/-}$;*p53*$^{-/-}$ tumors were with DMSO (ctrl), ZMC1, Olaparib or ZMC1+Olaparib. (B) Individual tumors (as in A) growth with corresponding treatment.

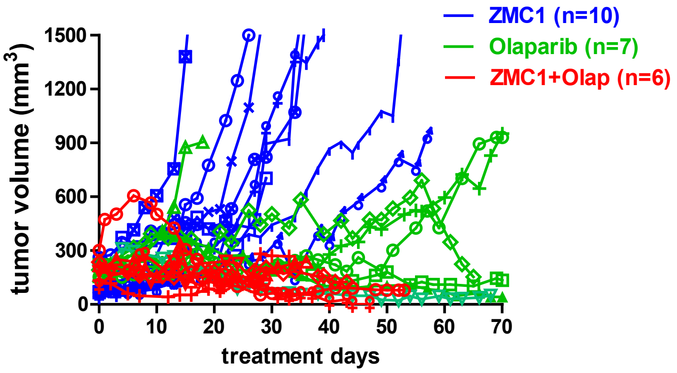

Supplementary Figure S6 Tumor-bearing *WAP-Cre;Brca1$^{f/f}$;p53$^{R172H/f}$* mammary tumor-bearing mice were treated with ZMC1 (2.5 mg/kg/day, n=10), olaparib (50 mg/kg/day, n=7), or ZMC1+olaparib (n=6).

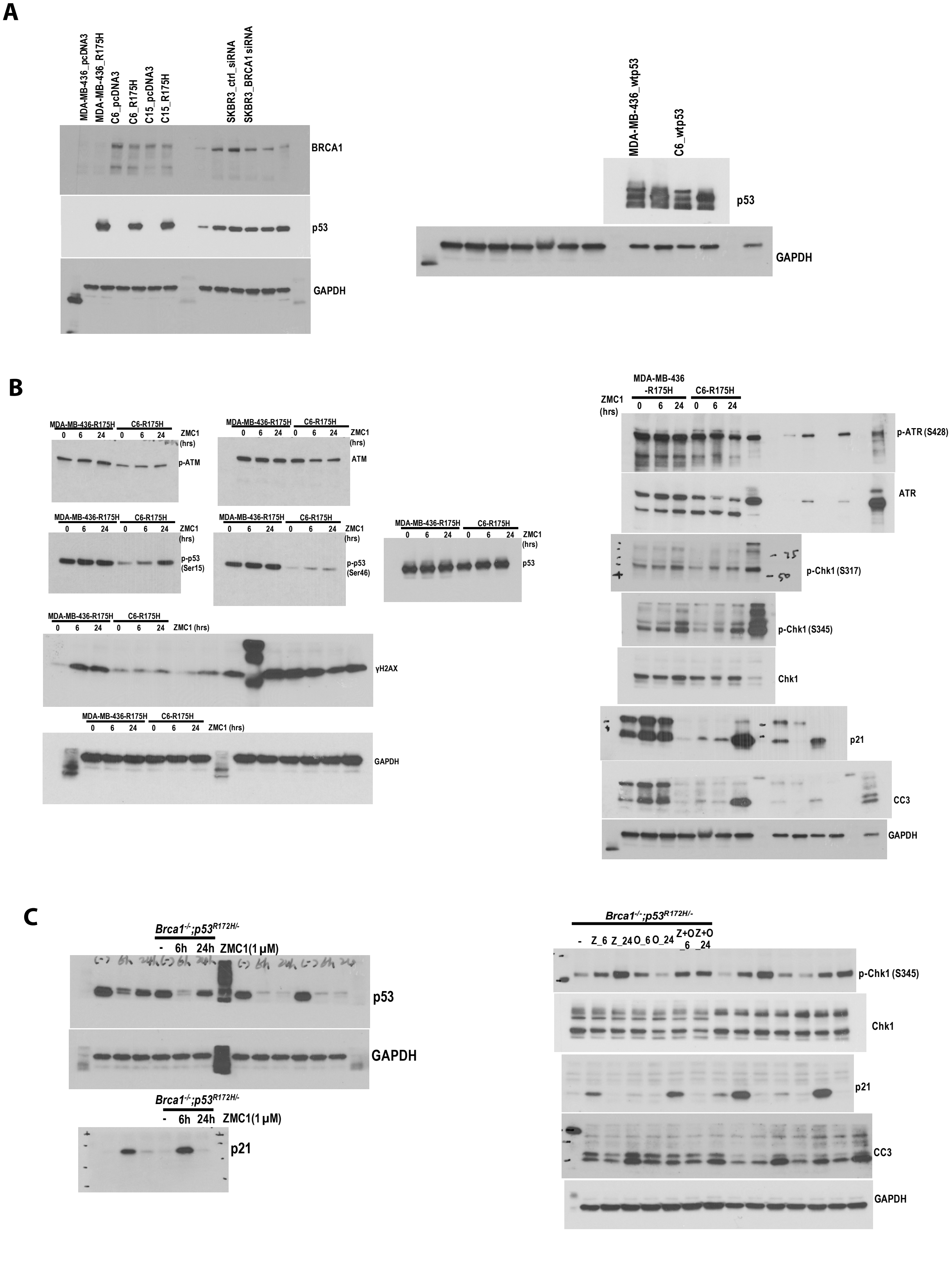

Supplementary Figure S7 Uncropped Western blots for Fig. 1c (A), Supplementary Figure S1B (A) and C (A), Fig. 1f (B), Fig. 2d (C) and Supplementary Figure S4A (C).
